# Influence of the *Saccharomyces cerevisiae*-based probiotic complex on gut microbiota, serum biochemistry, and circulating trace element and mineral levels in lactating dairy cows

**DOI:** 10.14202/vetworld.2024.1864-1871

**Published:** 2024-08-24

**Authors:** Elena A. Sizova, Elena V. Yausheva, Ksenia S. Nechitailo, Aina M. Kamirova, Kristina V. Ryazanceva, Daniil E. Shoshin, Anatoly V. Skalny, Alexey A. Tinkov

**Affiliations:** 1 Federal Research Centre of Biological Systems and Agrotechnologies of the Russian Academy of Sciences, Orenburg, Russia, 460000; 2 Scientific-Educational Center “Biological Systems and Nanotechnologies”, Orenburg State University, Orenburg, Russia, 460018; 3 Center for Bioelementology and Human Ecology, IM Sechenov First Moscow State Medical University (Sechenov University), Moscow, Russia, 119991; 4 Laboratory of Microbial Persistence and Symbiosis, Institute of Cellular and Intracellular Symbiosis, Russian Academy of Sciences, Orenburg, Russia, 460000

**Keywords:** gut microbiota, minerals, probiotic, *Saccharomyces cerevisiae*, trace elements

## Abstract

**Background and Aim:**

The existing data demonstrate that gut microbiota is involved in regulating mineral metabolism in cattle, although the data are quite contradictory. The study aimed to evaluate *Saccharomyces* *cerevisiae*-based probiotic’s effects on gut microbiota, systemic metabolism, and dairy cows’ essential trace element and mineral body burden.

**Materials and Methods:**

Fifteen cows received a daily supplement of a 50 g *S. cerevisiae*-based probiotic, fortified with methionine, choline, eugenol, cinnamaldehyde, and *Capsicum* oleoresin, for a month. 16S metagenomic sequencing was used to evaluate the taxonomic features of fecal microbiota. Serum trace elements and minerals levels were determined through inductively coupled plasma mass spectrometry.

**Results:**

Supplementation with *S. cerevisiae*-based probiotic complex significantly increased alpha and beta diversity, as well as the abundance of *Mediterranea* and *Clostridium IV* within the *Bacillota* phylum, whereas that of *Bacteroidota* and specifically *unclassified Bacteroidales* and *unclassified Oscillospiraceae* decreased. Following probiotic supplementation with the *S. cerevisiae*-based complex, gut microbiota modulation led to a significant boost in circulating levels of calcium, copper, selenium, and zinc. Creatinine levels decreased while total cholesterol levels increased within normal limits in the serum analysis.

**Conclusion:**

The observed improvement in trace elements and minerals in dairy cows might be due to changes in intestinal microflora caused by supplementation. Therefore, probiotic supplementation in cattle may be considered a potential tool for improvement of mineral nutrition in cattle. However, the influence of probiotic treatment and modulation of mineral metabolism on milk productivity and overall performance in cattle is yet to be estimated.

## Introduction

Gut microbiota plays a significant role in ruminant health because of its involvement in multiple metabolic processes [1] and subsequent modulation of organ functioning through gut-organ axes [2]. Specifically, the characteristics of gut microbiota are closely associated with immune response [3], and its alterations are involved in the pathogenesis of mastitis [4]. Gut microbiota is also involved in growth and development [5] and milk productivity [6].

Manipulating the gut microbiota in cattle, which influences both their health and milk productivity, is a proposed approach for improving their overall performance [7]. Probiotic products contain potentially beneficial microbial strains including *Bacillus, Enterococcus, Lactobacillus, Pediococcus*, and *Streptococcus* that were shown to be more effective in small livestock, whereas probiotic yeast (*Saccharomyces cerevisiae*) possesses more effect in adult ruminants [8]. Probiotic yeast supplementation enhances growth performance, feed efficiency, immunity, and metabolism [9].

The gut microbiota significantly influences nutrient metabolism [10]. Trace element and mineral absorption are regulated by gut microbiota biodiversity and characteristics, significantly impacting their metabolism [11]. Trace elements, such as zinc (Zn) [12] and selenium (Se) [13], also significantly affect the integrity of the gut microbiota and gut wall. Trace elements and minerals [14] are vital for managing metabolism and livestock health, whereas a deficiency [15] can cause diseases [16]. Milk productivity in dairy cows is influenced by their trace element intake [17]. The effects of altering gut microbiota on cattle well-being and productivity could potentially be attributed to shifts in trace element metabolism.

The study aimed to evaluate the effects of an *S. cerevisiae*-based probiotic complex on gut microbiota, systemic metabolism, and essential trace elements and minerals in dairy cows.

## Materials and Methods

### Ethical approval

The animal study protocol was approved by the Committee of Federal Research Centre for Biological Systems and Agrotechnologies of the Russian Academy of Sciences (No. 5 of 02/05/2021).

### Animal characteristics

A total of 15 healthy 5–6-year-old (body weight of 620.3 ± 15.3 kg) red steppe dairy cows were enrolled in the present study. Average milk production varied from 9.4 to 10.8 L/day. During the study, cows grazed on natural pastures predominantly formed by Kentucky bluegrass, bulbous bluegrass, Lessing feather grass, European feather grass, wild rye grass, common couch, Volga fescue, and meadow fescue. All animals had access to a salt lick block (99% NaCl) and drinking water *ad libitum*. During a month, the cows are supplemented (50 g/day) with an *S. cerevisiae*-based probiotic complex enriched with methionine, choline, eugenol, cinnamaldehyde, and *Capsicum* oleoresin (Rumenfit^®^, Mustang Feeding Technologies Ltd., Russia). Trace element and mineral levels in Rumenfit^®^ samples were evaluated prior to the study. The venous blood and fecal samples were collected before and after a month of probiotic supplementation.

### Sampling

Venous blood samples for blood count were collected from the tail vein using APEXLAB (Apexlab, Moscow, Russia) vacuum tubes with anticoagulant (ethylenediaminetetraacetic acid). Tubes with a clot activator (Hebei Xinl Sky & Tech Co., Ltd) were used for the collection of blood serum. Venous blood samples for trace element and mineral analyses were collected using VACUETTE^®^ CAT Serum Clot Activator Tubes (Greiner Bio-One International AG, Austria).

A total of 0.2–0.3 mL of feces were collected with a sterile instrument into a test tube with the preservative solution (DNA/RNA Shield, United States). The obtained samples were frozen at −60°C in Eppendorf tubes (Eppendorf, United States) until analysis.

### Analysis of the fecal microbiome

The obtained samples were homogenized with Lysing Matrix Y (MP Biomedicals, Solon, USA) using TissueLyser LT (Qiagen, Hilden, Germany). DNA extraction from the samples was performed using a QIAamp Fast DNA stool mini kit (Qiagen). The quality of DNA extraction was assessed using electrophoresis in 1% agarose gel and a Nanodrop 8000 (ThermoFisher Scientific, Waltham, MA, USA). DNA concentration was assessed with a double-stranded DNA high-sensitivity assay kit (Life Technologies, Carlsbad, CA, USA) using a Qubit 4 Fluorometer (Life Technologies,).

DNA libraries were prepared according to the Illumina protocol (Part #15044223, Rev. B.) with primers targeting the V3–V4 regions of the small subunit ribosomal RNA gene, S-D-Bact-0341-b-S-17 (5′-CCTACGGGNGGCWGCAG-3′) as the forward primer and S-D-Bact-0785-a-A-21 (5′-GACTACHVGGGTATCTAATCC-3′) as the reverse primer. The reaction mixture (25 μL) contained 0.2 μM of both primers and 80 μM deoxyribonucleotide triphosphates and 0.2 U Q5 high-fidelity DNA Polymerase (New England Biolabs, Ipswich, MA, USA). The prepared DNA libraries were cleaned using Agencourt AMPure XP beads (Beckman Coulter, Brea, CA, USA) and validated by capillary electrophoresis using the QIAxcel DNA screening kit (Qiagen). Paired-end 2 × 251 bp sequencing was performed with Reagent Kit v2 (Illumina, San Diego, CA, USA) using the MiSeq platform (Illumina, San Diego, CA, USA).

Initially, the raw reads obtained because of sequencing were evaluated using FastQC version 0.11.7 (Babraham Institute, UK). Paired-end reads were merged using USEARCH v10.0.240_win32 (drive5.com/usearch). After merging and adapter removal, the reads were re-evaluated using FastQC version 0.11.7. Subsequent treatment of merged reads was conducted using Usearch v10.0.240_win32 (drive5.com/usearch). Filtering quality was evaluated using FastQC version 0.11.7. Subsequently, rereplication and clustering of the filtered reads using the UPARSE algorithm were performed to identify operational taxonomic units (OTUs). Chimeric sequences were detected and subsequently removed using the UCHIME2 algorithm. Final OTUs were aligned to the initial merged reads using global alignment (usearch_global tool) at a similarity level of 97%. Taxonomic classification of sequences was conducted using the ribosomal database project reference database (rdp.cme.msu.edu/index.jsp). After filtering and assigning taxonomic affiliations, the resulting OTUs were used to calculate alpha (Chao1, Fisher’s alpha, Simpson, and Shannon2 indexes) and beta microbiota diversity using analysis of variance (ANOVA) and permutational multivariate analysis of variance (PERMANOVA), respectively.

### Blood count

Evaluation of red blood cell and white blood cells (WBC), platelet count, hemoglobin levels, and hematocrit was performed using a SysmexXS1000 (Sysmex, Kobe, Japan) hematology analyzer.

### Serum biochemistry

Assessment of serum total protein, total and direct bilirubin, creatinine, glucose, total cholesterol, urine, and uric acid levels, as well as alanine aminotransferase and aspartate aminotransferase activity, was performed using the respective Randox kits (Randox Laboratories Ltd., UK) at an automated biochemical analyzer CS-T240 (Dirui Industrial Co., Ltd., China).

### Trace element and mineral analysis

Before analysis, the obtained serum samples were subjected to pre-analytical treatment. Briefly, thawed samples were diluted at a volume ratio of 1:15 with a solution (pH = 2.0) consisting of 1% 1-Butanol (Merck, Germany), 0.1% Triton X-100 (Sigma-Aldrich, USA), and 0.07% HNO_3_ (Sigma-Aldrich) diluted in 18.2 MΩ cm distilled deionized water. Assessment of serum calcium (Ca), cobalt (Co), copper (Cu), iron (Fe), magnesium (Mg), manganese (Mn), selenium (Se), and zinc (Zn) concentrations was performed using inductively coupled plasma mass spectrometry at NexION 300D (PerkinElmer Inc., Shelton, CT, USA) equipped with a 7-port FAST valve and ESI SC-2 DX4 autosampler (Elemental Scientific Inc., Omaha, NE, USA). System calibration was performed using solutions prepared from Universal Data Acquisition Standards Kits (PerkinElmer Inc., Shelton, CT 06484, USA). Internal online standardization was performed using 10 μg/L solutions of Yttrium and Rhodium prepared from pure single-element standard kits (PerkinElmer Inc., Shelton, CT 06484, USA). Laboratory quality control was performed permanently using the certified reference materials blood plasma ClinChek^®^ Plasma Control (RECIPE Chemicals + Instruments GmbH, Germany) with recovery rates of 88%–107%.

### Statistical analysis

The obtained data were processed using Statistica 10.0 software (StatSoft, Tulsa, OK, USA). Data are expressed as mean and the respective standard deviation values. Comparative analysis of pre- and post-treatment values was performed using ANOVA with Bonferroni adjustment. The difference was considered significant at p < 0.05.

## Results

Analysis of the dairy cow gut microbiome revealed a high diversity of taxonomic groups. Specifically, 702007 sequencing reads were obtained using 18484–60820 reads per sample. After merging and filtration, 691813 sequencing reads were clustered into 2456 OTUs. Exclusion of singletons and doubletons and removal of potential sample contamination yielded 3329 OTUs. The obtained OTUs were grouped into specific taxa from phylum to genus (Table-1).

**Table-1:** A number of taxa identified in dairy cow intestinal microbiota.

Taxa	Pre-supplementation	Post-supplementation
Phylum	12 ± 3	12 ± 4
Class	22 ± 7	25 ± 8
Order	27 ± 8	36 ± 12
Family	45 ± 14	56 ± 19
Genus	95 ± 30	126 ± 44

Data are expressed as mean and the respective standard deviation

Analysis of the taxonomic characteristics of gut microbiota (Table-2) at baseline revealed a high prevalence of *Bacillota* and *Bacteroidota* phyla, accounting for 90% of all identified bacteria. The most common taxa were classes *Bacteroidia* and *Clostridia* and families *Oscillospiraceae, unclassified Bacteroidales, Lachnospiraceae* and *Bacteroidaceae*.

**Table-2:** Relative abundance (%) of taxa at the phylum, family, and genus levels in dairy cows before and after supplementation with *Saccharomyces cerevisiae*.

Taxa	Pre-supplementation	Post-supplementation	p-value
Phylum			
*Bacteroidota*	35.57 ± 3.06	29.39 ± 0.89	<0.001*
*Bacillota*	56.45 ± 3.07	64.31 ± 1.36	<0.001*
Other	7.98 ± 0.89	6.30 ± 0.73	0.980
Class			
*Bacteroidia*	35.54 ± 3.05	29.35 ± 0.89	<0.001*
*Clostridia*	54.01 ± 2.89	60.85 ± 1.45	<0.001*
*Negativicutes*	2.02 ± 0.21	1.75 ± 0.19	0.102
Other	8.44 ± 0.9	9.80 ± 0.76	0.790
Family			
*Bacteroidaceae*	9.44 ± 1.21	7.79 ± 0.21	<0.001*
*Unclassified Eubacteriales*	2.38 ± 0.66	2.09 ± 0.07	<0.001*
*Rikenellaceae*	4.45 ± 0.52	3.47 ± 0.21	<0.001*
*Unclassified Bacteroidales*	18.01 ± 1.72	15.17 ± 0.45	<0.001*
*Lachnospiraceae*	11.43 ± 2.40	11.00 ± 0.001	0.002
*Oscillospiraceae*	36.83 ± 0.001	42.66 ± 1.56	<0.001*
Other	17.46 ± 1.06	8.05 ± 0.7	0.769
Genus			
*Phocaeicola*	6.46 ± 0.95	5.59 ± 0.22	<0.001*
*Alistipes*	3.18 ± 0.35	2.97 ± 0.17	<0.001*
*Mediterranea*	2.38 ± 0.33	15.17 ± 0.45	<0.001*
*Unclassified Bacteroidales*	18.01 ± 1.72	1.79 ± 0.08	<0.001*
*Unclassified Lachnospiraceae*	8.65 ± 2.05	11.00 ± 0.51	0.053
*Clostridium IV*	2.30 ± 0.24	39.6 ± 1.64	0.059
*Unclassified_Oscillospiraceae*	29.91 ± 0.91	2.09 ± 0.07	<0.001*
*Unclassified Eubacteriales*	2.38 ± 0.20	0.73 ± 0.04	<0.001*
Other	26.74 ± 0.98	25.35 ± 0.77	0.986

Data are expressed as mean and the respective standard deviation,

* Significant difference in comparison to pre-supplementation values according to one-way analysis of variance with Bonferroni adjustment

Supplementation with *S. cerevisiae* resulted in significant changes in the taxonomic composition of gut microbiota. Specifically, at the end of the supplementation period, the abundance of *Bacteroidota* in the fecal microbiome was 6% lower, whereas that of *Bacillota* was 8% higher than that at baseline. In addition, certain taxa within these phyla were also affected by *S. cerevisiae* supplementation. In particular, 13% and 37% increases in the abundance of *Mediterranea* and *Clostridium IV* were observed after supplementation, respectively. At the same time, the abundance of *unclassified Bacteroidales* and *Oscillospiraceae* was characterized by a significant 16% and 28% decrease compared with the baseline values.

Assessment of bacterial community diversity using Chao1 and Fisher’s α index revealed higher bacterial biodiversity in dairy cows after supplementation with *S. cerevisiae* as compared to the baseline. At the same time, no significant group difference in Shannon_2 and Simpson indices between pre- and post-supplementation periods was observed (Table-3).

**Table-3:** Gut microbiota α biodiversity indices in dairy cows before and after supplementation with *Saccharomyces cerevisiae*.

Diversity index	Pre-supplementation	Post-supplementation	p-value
Chao1	548.6 ± 19.4	840.4 ± 43.3	<0.001*
Fisher’s alpha	100.3 ± 4.33	140.5 ± 8.08	0.001*
Simpson	0.99 ± 0.002	0.98 ± 0.003	0.048*
Shannon_2	5.24 ± 0.08	5.24 ± 0.09	0.958

Data are expressed as mean and the respective standard deviation,

* Significant difference in comparison to pre-supplementation values according to one-way analysis of variance with Bonferroni adjustment

**Figure-1:**
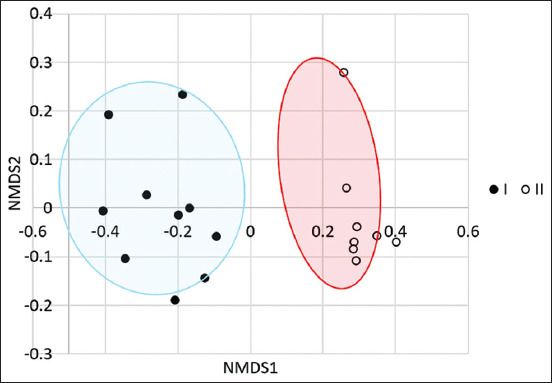
Nonmetric multidimensional scaling plot of beta diversity of intestinal microbiota in dairy cows before (filled circles) and after (empty circles) supplementation with *Saccharomyces cerevisiae*-based probiotic complex.

PERMANOVA analysis of β-biodiversity (Figure-1) revealed significant group differences between the pre- and post-supplementation periods in dairy cows (F-value: 7.8542; p-value: 0.001).

Despite a significant impact on gut microbiota in dairy cows, *S. cerevisiae*-based probiotic complex supplementation did not result in significant changes in blood count, although a non-significant trend toward increased WBC numbers was observed (Table-4).

**Table-4:** The impact of *Saccharomyces cerevisiae*-based probiotic complex on blood count in dairy cows.

Parameter	Pre-supplementation	Post-supplementation	p-value
WBC, 10^9^/L	10.582 ± 4.864	13.66 ± 5.811	0.215
RBC, 10^12^/L	5.196 ± 0.453	5.217 ± 0.879	0.947
Hb, g/L	106.4 ± 11.64	103.3 ± 15.17	0.614
Ht, %	25.31 ± 2.541	23.68 ± 4.501	0.332
PLT, 10^9^/L	232 ± 54.02	276.2 ± 103.89	0.248

Data are expressed as mean and the respective standard deviation, RBC=Red blood cell, WBC=White blood cell, PLT=Platelet, Hb=Hemoglobin, Ht=hematocrit

In contrast to blood count, *S. cerevisiae*-based probiotic complex had a significant effect on blood biochemistry (Table-5). Specifically, total cholesterol levels in the post-supplementation period exceeded the respective baseline values by 27%. At the same time, the post-supplementation values were found to fit the reference ranges. In contrast, circulating creatinine concentrations were significantly decreased by 19% compared with the pre-supplementation period.

**Table-5:** Serum biochemistry in dairy cows before and after supplementation with *Saccharomyces cerevisiae*-based probiotic complex.

Parameter	Pre-supplementation	Post-supplementation	p-value
Glucose, mmol/L	3.327 ± 0.384	3.29 ± 0.434	0.842
Total protein, g/L	94.53 ± 7.74	89.13 ± 12.98	0.274
ALT, U/L	29.42 ± 4.99	32.48 ± 9.32	0.372
AST, U/L	81.2 ± 22.05	88.7 ± 34.81	0.572
Total bilirubin, μmol/L	1.784 ± 1.853	1.477 ± 0.478	0.618
Direct bilirubin, μmol/L	1.266 ± 0.775	1.244 ± 0.234	0.932
Total cholesterol, mmol/L	3.153 ± 0.711	4.009 ± 0.82	0.023*
Urea, mmol/L	3.69 ± 1.414	3.81 ± 1.414	0.852
Creatinine, μmol/L	131.92 ± 15.54	106.26 ± 19.46	0.004*
Uric acid, μmol/L	35.11 ± 9.051	41.04 ± 11.312	0.212

Data expressed as mean and the respective standard deviations,

* Significant group differences at *P* < 0.05 following analysis of variance with Bonferroni adjustment, ALT=Alanine aminotransferase, AST=Aspartate aminotransferase

The levels of calcium (Ca), cobalt (Co), copper (Cu), iron (Fe), magnesium (Mg), Se, and Zn in probiotic samples were 21374 ± 687, 13.8 ± 2.1, 90.1 ± 22.7, 633.7 ± 10.1, 4963 ± 483, 1,134 ± 0,171, 441.5 ± 71.6 µg/g, respectively. Supplementation with the *S. cerevisiae*-based probiotic complex had a more profound impact on serum trace element and mineral levels (Table-6). Specifically, the post-supplementation concentrations of Ca, Cu, Se, and Zn significantly exceeded the pre-supplementation values by 6, 38, 14, and 18%, respectively. At the same time, nutritional intervention did not affect the levels of Co, Fe, Mg, and Mn in the serum of dairy cows.

**Table-6:** Serum trace element and mineral levels in cows supplemented with *Saccharomyces cerevisiae*-based probiotic complex.

Element	Pre-supplementation	Post-supplementation	p-value
Ca, μg/mL	99.74 ± 6.56	105.73 ± 4.51	0.035*
Co, ng/mL	1.134 ± 0.547	0.807 ± 0.82	0.308
Cu, μg/mL	0.638 ± 0.142	0.822 ± 0.169	0.017*
Fe, μg/mL	2.819 ± 1.493	2.829 ± 0.523	0.984
Mg, μg/mL	24.19 ± 3.82	25.06 ± 1.73	0.519
Mn, ng/mL	3.528 ± 1.798	3.47 ± 0.536	0.923
Se, μg/mL	0.087 ± 0.016	0.099 ± 0.005	0.028*
Zn, μg/mL	0.99 ± 0.203	1.166 ± 0.155	0.043*

Data expressed as mean and the respective standard deviations,

* Significant group differences at *P* < 0.05 following analysis of variance with Bonferroni adjustment, Zn=Zinc, Se=Selenium, Ca=Calcium, Co=Cobalt, Cu=Copper, Fe=Iron, Mg=Magnesium, Mn=Manganese

Despite a significant impact on gut microbiota in dairy cows, *S. cerevisiae*-based probiotic complex supplementation did not result in significant changes in blood count, although a non-significant trend toward increased WBC numbers was observed (Table-4).

## Discussion

The probiotic complex made up of *S. cerevisiae* and supplemented with methionine, choline, eugenol, cinnamaldehyde, and *Capsicum* oleoresin significantly increases intestinal microbiota biodiversity, boosts essential trace element and mineral levels in serum, lowers creatinine, and raises total cholesterol levels within acceptable limits.

### Effects on gut microbiota

The effect of the *S. cerevisiae*-based supplement on intestinal microbiota aligns with previous research findings. Supplementing *S. cerevisiae* improved the rumen microbiota [18]. Probiotic *S. cerevisiae* is suggested to enhance gut wall integrity, boost intestinal immunity, lessen proinflammatory cytokine production, and obstruct pathogenic bacteria attachment to the gut wall [19]. Probiotic yeast has significantly reduced the abundance of Bacteroidetes in cattle rumen [20]. An increase in both *Clostridium* XlVb and *Oscillibacter* abundance was noted in mice after yeast protein supplementation [21].

In addition to *S. cerevisiae*, other constituents of the complex may have a significant modulating effect on gut microbiota. Specifically, choline was shown to increase gut microbiota alpha diversity, whereas an increase in beta diversity was observed for *Firmicutes, Proteobacteria*, and *Actinobacteria*, whereas the abundance of *Bacteroidetes, Spirochaetes*, and *Euryarchaeota* was reduced in gilts [22]. Methionine supplementation in heifers significantly increased the abundance of *Geodermatophilaceae, Nocardioidaceae, Amycolatopsis*, S24_7, *Clostridiales, Novosphingobium, Anaeroplasma*, and *Anaeroplasmatales*, while decreasing the abundance of *Dietzia, Dietziaceae, Collinsella, Coriobacteriales, Barnesiellaceae, Staphylococcaceae, Pseudoramibacter*_*Eubacterium*, and *Anaerotruncus* [23]. Similarly, eugenol was shown to possess a significant modulatory effect on gut microbiota by inhibiting the growth of certain pathogenic bacteria, including *Listeria monocytogenes* and *Clostridium difficile* to name a few [24]. In cattle, eugenol supplementation reduces the number of intestinal coliform bacteria and improves lactate production [25]. Cinnamaldehyde also has an antibacterial effect against bacterial pathogens [26]. Specifically, trans-cinnamaldehyde was shown to reduce *Escherichia coli* growth [27]. It has also been demonstrated that *Capsicum* oleoresin treatment significantly reduced the whole ruminal contents of *Bacteroidales* and increased that of *Bifidobacteriales* in lactating dairy cows [28].

### Effects on trace element and mineral metabolism

Probiotics can cause an increase in the body’s retention of trace elements and minerals in livestock by reducing their excretion [29]. It is proposed that healthy gut microbiota may promote micronutrient bioavailability through the improvement of digestion processes and production of short-chain fatty acids that maintain acidic reaction at the luminal side of the gut wall, thus promoting the uptake of certain trace elements like Fe2+ [30]. Providing *S. cerevisiae* CNCM I-1077 to finishing bulls enhances their serum Zn and Cu levels, along with hepatic Cu storage, yet decreases muscular Fe content [31]. Increasing the cyc-methionine dose to 2 g/day for growing Awassi lambs led to elevated hepatic and muscular Zn, Cu, and Co levels, while a 4 g/day dosage showed no effect [32]. Supplying Hanson steers with a combination of *Bacillus* and *Saccharomyces* cultures led to a considerable increase in P, Mg, K, Na, Fe, and Zn content within the longissimus muscle [33]. The ability of yeast supplementation to improve trace elements and minerals absorption might be attributed to its effects on fiber digestion and stabilizing rumen pH [34]. In addition, other components in the complex can significantly influence trace element metabolism. Specifically, it has been demonstrated that cinnamaldehyde treatment is also capable of modulating trace element metabolism in *Caenorhabditis elegans* by increasing Mn and Fe content [35], although these metals were unaffected by supplementation in the present study.

If the used supplements were not enriched with trace elements and minerals, the observed changes in serum trace element and mineral levels after supplementation with an *S. cerevisiae*-based probiotic could be mediated by the modulation of gut microbiota.

Modulating gut microbiota can influence trace element metabolism significantly by altering bioavailability and absorption in the host [11]. These taxa – *Bacteroides, Oscillibacter, Dialister, Clostridium, Lactobacillus*, and *Streptococcus salivarius* – enhance Ca bioavailability, while *Lactobacillus acidophilus* and Bifidobacteria facilitate Zn absorption. *Streptococcus thermophilus, Enterococcus faecium, Bifidobacterium longum*, and *Lactiplantibacillus plantarum* increase Se bioavailability [30].

### Effects on metabolism

Dairy cows given an *S. cerevisiae*-based probiotic complex experienced a significant decrease in circulating creatinine levels, suggesting improved kidney function. Earlier studies revealed a decrease in circulating creatinine levels in Holstein cows after probiotic intake [36]. Supplementation of *S. cerevisiae* led to a decrease in creatinine levels in Holstein cows [37]. Other constituents in the complex may also contribute to its nephroprotective effect. Eugenol’s antioxidant activity, accountable for its protective effect against kidney damage, is due to the activation of nuclear factor erythroid 2-related factor 2 (Nrf2) signaling [38]. Cinnamaldehyde has also been shown to reduce kidney senescence [39] and inflammation [40].

The study yielded intriguing results about the impact of *S. cerevisiae*-based probiotic complex on serum cholesterol. A significant increase in total cholesterol was noted in dairy cows at the end of the supplementation period. The values obtained did not surpass the standard dairy cow reference values [41]. Studies generally show that *S. cerevisiae* supplementation lowers cholesterol levels in livestock [9]. Reportedly, some studies have identified an augmentation in cows’ plasma cholesterol after *S. cerevisiae* supplementation [42]. It is proposed that the observed increase in total cholesterol levels may be associated with the activation of cholesterol biosynthesis in dairy cows, which is physiologically involved in the functioning of the reproduction system [43]. Serum cholesterol was found to correlate significantly with daily milk yield [44], being higher in cows with high milk yield [45]. Circulating cholesterol increases with Cu supplementation [46], so total cholesterol increase may result from this.

## Conclusion

The intake of *S. cerevisiae*-based probiotics fortified with methionine, choline, eugenol, cinnamaldehyde, and *Capsicum* oleoresin resulted in increased gut microbiota richness. It improved essential trace elements and minerals in the bloodstream. After supplementation, *Bacteroidota* abundance decreased while *Bacillota* abundance increased, leading to an increase in fecal alpha and beta diversity. Supplementation with the *S. cerevisiae*-based probiotic complex led to a significant increase in circulating levels of Ca, Cu, Se, and Zn that were linked to a modulated gut microbiota. The absence of additional trace elements in the supplement and the role of gut microbiota in micronutrient absorption suggest that the improvement in trace element and mineral status in dairy cows may be due to supplementation-induced changes in gut microflora.

## Data Availability

The datasets generated during the current study are available from the corresponding author on reasonable request.

## Authors’ Contributions

EAS and AAT: Conceptualization and data curation. EVY, KSN, AMK, KVR, and DES: Methodology. AVS: Validation. AAT: Formal analysis and writing – original draft preparation. EAS, EVY, KSN, AMK, KVR, and AAT: Investigation. EAS and AVS: Writing – review and editing. All authors have read and agreed to the published version of the manuscript.
